# Imaging of Ulcerative Colitis: The Role of Diffusion-Weighted Magnetic Resonance Imaging

**DOI:** 10.3390/jcm13175204

**Published:** 2024-09-02

**Authors:** Ali S. Alyami

**Affiliations:** Department of Diagnostic Radiography Technology, College of Nursing and Health Sciences, Jazan University, Jazan 45142, Saudi Arabia; aalmansour@jazanu.edu.sa

**Keywords:** diffusion, apparent diffusion coefficient, ulcerative colitis, MRI, disease activity

## Abstract

Magnetic resonance imaging (MRI) has emerged as a promising and appealing alternative to endoscopy in the objective assessment of patients with inflammatory bowel disease (IBD). Diffusion-weighted imaging (DWI) is a specialized imaging technique that enables the mapping of water molecule diffusion within biological tissues, eliminating the need for intravenous gadolinium contrast injection. It is expanding the capability of traditional MRI sequences in Ulcerative Colitis (UC). Recently, there has been growing interest in the application of intravoxel incoherent motion (IVIM) imaging in the field of IBD. This technique combines diffusion and perfusion information, making it a valuable tool for assessing IBD treatment response. Previous studies have extensively studied the use of DWI techniques for evaluating the severity of activity in IBD. However, the majority of these studies have primarily focused on Crohn’s disease (CD), with only a limited number of reports specifically examining UC. Therefore, this review briefly introduces the basics of DWI and IVIM imaging and conducts a review of relevant studies that have investigated its application in UC to show whether these techniques are useful techniques for evaluating patients with UC in terms of detection, characterization, and quantification of disease activity. Through the extensive literature survey, most of these studies indicate that DWI proves valuable in the differential diagnosis of UC and could be used as an effective modality for staging UC.

## 1. Introduction

Ulcerative colitis (UC) is a chronic idiopathic inflammatory condition of the colonic and rectal mucosa, with continuous extending and a predictable course, as well as localisation that spreads from the rectal tract proximally to include different portions of the colon (pancolitis or left-side colitis sigmoiditis and ulcerative proctitis) [1]. Chronic inflammation causes structural damage to the organ, resulting in reduced function and impairment. In patients with UC, fibrosis is categorised by the thickening of the muscularis mucosae and the excessive deposition of the extracellular matrix (ECM) within the submucosa [2]. This pathological process primarily impacts deeper layers subsequent to significant ulceration of the submucosal tissue. However, recent findings indicate that fibrous deposition within the submucosa layer was observed in all colectomy specimens obtained from patients with UC who underwent surgery due to refractory disease [3]. Strictures are infrequent occurrences in UC, with the vast majority being benign in nature and potentially reversible [4,5]. Yet, patients diagnosed with UC exhibit impaired colonic motility, indicating a dysfunction in the movement and contraction of the colon, and elevated ECM levels and subsequent stiffening of the colonic wall can contribute to aberrant colonic motility, causing such clinical manifestations as frequent bowel movements, loose stools or diarrhoea, pain or abdominal discomfort, and even in the absence of inflammatory processes [2,3,6]. The available evidence on fibrosis in UC is currently limited and subject to controversy. However, a thorough evaluation conducted by Gordon et al. in 2018 provided comprehensive insights, revealing that UC is associated with progressive fibrosis and muscularis mucosa thickening, which exhibit a correlation with the severity and chronicity of inflammation [3]. Therefore, deep remission, which includes histological remission, is an essential therapeutic target in the management of UC [7]. Furthermore, patients with UC face an elevated risk of developing colorectal cancer, particularly when the disease has a long duration, is associated with primary sclerosing cholangitis and involves chronic inflammation [8]. 

Conventional colonoscopy, despite being considered the gold standard for assessing disease activity and severity in patients with UC, has drawbacks, mostly due to its invasiveness and requirement of colonic preparation. Patients also find it less acceptable than other UC monitoring techniques [9]. While endoscopy and laboratory tests offer valuable insights into the severity and extent of inflammation in the gastrointestinal tract, their utility in assessing the response to biological therapy is limited due to several constraints. Mucosal healing is a crucial therapeutic goal in UC, associated with better outcomes, such as reduced relapse rates, a lowered colorectal cancer risk and decreased hospitalisation rates [10,11]. However, there are currently no validated definitions of mucosal healing and no standardised criteria for evaluating disease activity. In the most recent update of the Selecting Therapeutic Targets in Inflammatory Bowel Disease (STRIDE-2) programme, it is recommended that achieving an Ulcerative Colitis Endoscopic Index of Severity (UCEIS) ≤1 or a Mayo Endoscopic Subscore (MES) of zero should be recognised as indicators of endoscopic healing and serve as treatment targets for UC [12].

Non-invasive approaches, such as cross-sectional imaging, have been studied in patients with IBD [13], and such techniques as computed tomography (CT) and magnetic resonance enterography (MRE) can be used to assess disease activity and the response to biological therapy in IBD [14,15], as well as to detect complications of the disease, such as strictures or fistulae and abdominal abscesses [16]. However, these techniques require colonic preparation, like conventional colonoscopy [17,18]. DWI is a magnetic resonance imaging (MRI) technique that examines the movement of water molecules within cellular and extracellular spaces. Classified as a fast functional MRI, this technique is widely accepted for imaging IBD due to its reliance on molecular motion, allowing the visualisation of inflammatory lesions associated with IBD [19]. Moreover, DWI is particularly suitable for paediatric patients due to its ability to acquire images rapidly [20]. In the context of endoscopy or MR examinations, patients commonly experience poor tolerance to oral and rectal bowel cleansing preparations. However, the utilisation of DWI obviates the need for oral or rectal preparation and fasting. Moreover, the duration of the procedure was relatively brief, with the entire examination, including patient setup, routine MRI and DWI, taking approximately 20 min [21]. 

Previous studies have highlighted the utility of DWI in assessing inflammatory activity in UC. Specifically, the apparent diffusion coefficient (ADC) value has been found to exhibit an inverse correlation with the degree of inflammatory changes, indicating its potential as a valuable parameter for assessing inflammatory activity in UC. However, limited studies have demonstrated the value of DWI in inflamed portions of the colon in UC patients [22,23,24,25]. Therefore, this review aims to discuss the role of conventional DWI and its advancements and to consider current and future clinical applications of DWI to UC in terms of diagnosis, surveillance and treatment guidance. A comprehensive review of the literature available on this topic was performed. The literature search terms contained ‘Diffusion-weighted imaging’ OR ‘DWI’ and ‘intravoxel incoherent motion’ OR ‘IVIM’ combined with ‘ulcerative colitis’ OR ‘UC’. All relevant papers in English from the Ovid, PubMed and EMBASE databases were reviewed.

## 2. Overview of the DWI Method and Its Advancements

MRI employs powerful magnetic fields and radiofrequency waves to produce detailed images of the internal structures within the body. Meanwhile, DW-MRI is a technique that examines the natural, random movement of water protons in the body by generating an image contrast via the detection of variations in the diffusion of water molecules within different tissues. These protons in the body can be found in both intracellular and extracellular spaces. The free diffusion of water molecules is influenced by such factors as the bulk capillary flow and interactions with macromolecules and cell membranes. Therefore, DW-MRI offers valuable information about the cellular structure and organisation at a microscopic level. In a setting with no constraints, water movement would appear completely random, much like how molecules move in Brownian motion or freely diffuse. Whenever the environment is highly cellular, water diffusion is restricted, and the diffusion signal is enhanced. For example, in tissues with a high cellular density, such as tumour tissue, the extracellular space is characterised by increased tortuosity and a higher cell membrane density, resulting in the hindered apparent diffusion of water (reduced diffusion). Conversely, in necrotic and cystic tissues, where cellular barriers are diminished, water diffusion encounters fewer obstacles, leading to relatively unrestricted apparent diffusion [26,27].

This technique utilises magnetic field gradients and spin-echo MR sequences to investigate minute molecular motions, typically ranging from 20 to 30 µm, within the typical timescale of 50 to 100 MS in DWI. In an unrestricted environment, such as necrotic tissues or free fluid, tissue water molecules demonstrate high diffusion. However, in the context of solid tumours and inflammation, the increased cellular density leads to a reduction in the extracellular space and an increase in the intracellular volume. Consequently, this alteration results in restricted diffusion. DWI entails a relatively brief acquisition time and eliminates the need for intravenous contrast administration. Furthermore, it offers the advantage of providing both quantitative and qualitative imaging assessments. The quantitative evaluation of DWI involves the calculation of ADC, which necessitates the utilisation of at least two b values, where a b value is a factor that indicates the timing and strength of the gradients used to generate DWI.

The sensitivity of DW-MRI to water diffusion can be changed by adjusting a parameter called the b value, which is determined and proportional by different factors, such as the strength and duration of the applied two gradients and the interval between these gradients. By modifying the b value, the DW-MRI sequence can be optimised to enhance or attenuate the signal related to water diffusion, allowing for better visualisation and characterisation of tissue properties. Both high (800–1000 s/mm^2^) and very low (0–50 s/mm^2^) b values allow a high contrast-to-noise ratio and adequate ADC calculation [28,29]. Meanwhile, the qualitative DWI tissue assessment relates to different signal intensities because of water diffusion. In simple terms, when diffusion is unrestricted, it causes a decrease in the signal on DWI and higher values. This indicates areas with fewer cells where water can move more easily. Conversely, restricted diffusion results in an increased signal on DWI and lower ADC values, suggesting areas with more cells where water movement is constrained. Consequently, ADC and DWI values deliver a quantitative assessment of cellularity at the molecular level.

To generate ADC maps, data are acquired from two or more b values (typically three) [30]. The signal intensity (or logarithm of the signal intensity) from each voxel in the MR image is plotted against the corresponding b value to create a graph. The slope of this line represents the ADC value for that specific voxel. The ADC is calculated using the mathematical Equation (1):

(1) $$ADC=\frac{ln(SI_{0}/SI)}{b}$$

where SI_0_ refers to the signal intensity at b = 0, and SI represents the signal intensity at a higher b value. This process is automated for all voxels within the MR image, resulting in the creation of a parametric ADC map. The utilisation of ADC maps enables radiologists to discriminate between benign and malignant tissues, evaluate the aggressiveness of tumours and track temporal changes in the tissues.

Recently, advancements in the DWI technique allow more information to be obtained for lesion characterisation and detection by using DWI with multiple b-values or ultra-high b-values. The emergence of DWI has facilitated the introduction of novel water diffusion-derived metrics, such as intravoxel incoherent motion (IVIM). The theoretical principles of IVIM imaging have been thoroughly explained in prior studies [31,32], and a summary of the fundamental principles of IVIM will be provided in this review. In this sequence, a voxel is characterised by the presence of both intravascular and extravascular signal sources. Consequently, this results in the existence of two distinct diffusion coefficients: D*, which denotes the pseudo-diffusion coefficient that reflects the intricate motion of water within the intravascular networks, and D, which represents the molecular diffusion coefficient attributed to Brownian motion within the extravascular spaces [31,32]. As revealed by Le Bihan et al. [31], employing very low b-values (ranging from 0 to 200 s/mm^2^) allows for the detection of tissue micro-perfusion’s contribution to water diffusion. Despite the conceptualisation dating to the 1980s, technical constraints impeded its realisation until recent advancements. The evolution of MRI systems has now made it feasible to acquire multiple low b-values, thus enabling the clinical application of the IVIM technique. This technique does not require contrast agents, as it is based on perfusion MRI. Furthermore, IVIM-DWI enables the differentiation and assessment of perfusion and true molecular diffusion components through the estimation of various parameters, such as perfusion fraction (f), the pseudo-diffusion coefficient (D_fast) and the true diffusion coefficient (D_slow). This is accomplished by acquiring multiple b-values, typically equal to or greater than four, and employing a bi-exponential model to analyse the data. IVIM imaging offers a comprehensive approach to characterising tissue microcirculation and diffusion properties, providing valuable insights into the pathophysiology of various diseases, especially useful in pathologies with notable tissue perfusion, such as inflammatory or neoplastic conditions [33].

### Advantages of DWI in IBD

There are several advantages of using DWI in the field of medical imaging, as follows:

1. Bowel preparation is not required for the detection of colonic inflammation using DWI [24]. While most magnetic resonance colonography (MRC) protocols typically involve colonic distension using water or contrast enemas, DWI-MRC stands out as an exception, as it does not require colonic distension [34].
2. There is no need for intravenous contrast material. The main advantage of DWI in comparison to conventional MRI is its ability to detect bowel wall inflammation without the need for intravenous contrast material [24].
3. Unlike endoscopy, which can be uncomfortable and invasive, DWI uses MRI to assess disease activity without exposure to ionising radiation [35].
4. DWI offers a distinctive advantage in its capacity to evaluate tissue cellularity and microstructural integrity. Through the analysis of water molecule diffusion, MRI can provide valuable information regarding the cellular environment within tumours. This enables differentiation among various tissue types and facilitates the early detection of therapeutic response-related changes. The functional imaging capability of DWI complements conventional anatomical imaging, thereby enabling a more comprehensive assessment of different diseases, including IBD.
5. DWI can be combined with other MRI sequences to provide complementary information. For example, combining DWI with T2-weighted sequences allows a high degree of diagnostic accuracy for perianal fistulae, eliminating the need for gadolinium-based contrast media [36,37].

Conversely, there are certain limitations associated with the use of DWI-MRI in the assessment of IBD, including UC. For example, the limited depiction of anatomical details in DWI-MRI restricts the ability to evaluate comprehensively complications arising from intra-abdominal diseases [38]. Moreover, the role of DWI-MRI in evaluating the inflammatory activity in perianal fistulae remains uncertain [38].

## 3. The Role of MRI in UC Detection and Management

MRI is a highly effective imaging technique for comprehensively examining the entire gastrointestinal tract, making it a crucial tool for gastrointestinal radiologists involved in the study of IBD [39]. The inherent characteristics of MRI, including its superior contrast resolution, diverse imaging parameters, notable sensitivity of T2-weighted sequences to detect inflammation, ability to manipulate signals through fat suppression and remarkable enhancement achieved with gadolinium injection, establish MRI as a dependable diagnostic modality for IBD. MRI can identify most characteristic findings associated with these diseases and their related complications, and it enables the evaluation of the extent and severity of disease, even when fibrotic strictures hinder complete endoscopic assessments of the entire colon or in cases of severe acute disease, when endoscopy may be incomplete or contraindicated.

The significant role of MRE in the diagnosis, assessment of disease activity, identification of complications and evaluation of treatment response in Crohn’s disease (CD) has been widely acknowledged [40]. A recent systematic review and meta-analysis has provided evidence of the diagnostic accuracy of MRE in accurately assessing colonic abnormalities in individuals with CD [41]. Although endoscopy remains the primary modality for evaluating UC due to its ability to visualise the mucosal layer directly and to obtain tissue biopsies [42], MRE provides valuable insights into the involvement of deeper layers and inaccessible sites, thereby offering valuable information regarding extramural pathological conditions in UC [43,44]. Furthermore, MRE enables the detection of extraintestinal manifestations associated with UC [45]. In UC cases, initial endoscopic manifestations involve granularity, hyperaemia and mucosal oedema, which may contribute to the blunting of the colonic haustra [46]. Distinct characteristics of acute UC on MRE may manifest initially as mucosal or inner-wall hyperenhancement during the early phase. However, progressive inflammation subsequently gives rise to submucosal oedema, mucosal ulcerations and colonic wall thickening [47]. 

In the recent literature, the MRE descriptors of bowel wall inflammation involves pericolic lymphadenopathies, mural ulceration, mural oedema, diffusion restriction, mucosal hyperenhancement and an engorged pericentric mesentery [24,34,48]. Several studies have been conducted to investigate the accuracy of non-invasive MRI methods for the evaluation of UC activity [24,34]. For example, in patients with UC, Ords et al. [34] reported the sensitivity and specificity (87% and 88%, respectively) for identifying endoscopic inflammation by using a simplified index MRC (MRC-S ≥ 1) originating from MRC, which included enlarged lymph nodes, mural oedema, relative contrast enhancement and the ‘comb sign’. The findings of the study demonstrate that MRC exhibits a high accuracy when it comes to evaluating disease activity and severity in patients diagnosed with UC [34]. In cases of quiescent UC, it is possible to observe reduced mural thickening and the absence of hyperenhancement on imaging. According to reports, approximately 10% of patients with confirmed quiescent UC may exhibit normal MRE findings [49]. Current evidence suggests that MRE may not possess the capability to differentiate accurately between a mild and moderate MES or between a mild and moderate UCEIS score; thus, it is important to note that additional research is required to validate these findings.

In cases of fibrosis in chronic UC, there has been a significant lack of exploration and investigation into the endoscopic characterisation of fibrosis. Gordon et al. [3] identified submucosal fibrosis in all colectomy specimens from patients with UC. However, this fibrosis was observed exclusively in areas affected by inflammation. The presence of substantial fibrosis and muscularis mucosa thickening, which often goes undetected through endoscopic mucosal sampling, should be acknowledged as a frequent complication of chronic progressive UC [3]. 

Further, submucosal fat deposition is a characteristic indicator of chronic involvement, primarily observed in the rectal region, and it can be identified using MRE. The significance of utilising fat-suppressed T2-weighted sequences in distinguishing submucosal fat from oedema is of paramount importance. While fibrofatty proliferation is commonly observed as a characteristic feature of CD, pericolic fat proliferation can often be observed in patients with chronic UC during MRE, particularly in the perirectal and peri-sigmoid regions [50]. In cases of long-standing UC, a combination of fibrosis, submucosal fat accumulation, muscle hypertrophy and pericolic fat expansion collectively leads to luminal narrowing. Thus, MRE holds particular significance in patients presenting with undiagnosed colitis, as it could reveal imaging characteristics of chronicity that may not be captured through endoscopy [47].

Indications for considering MRE in patients with UC include specific clinical scenarios in which MRE can provide valuable information. UC primarily affects the colonic mucosa, which is typically fully accessible for evaluation using colonoscopy. Consequently, radiologists have historically utilised advanced imaging techniques less frequently in the management of UC compared to their application in CD. MRC was initially introduced as a method for evaluating colonic polyps and inflammation. However, its application is limited due to low patient acceptance and the superior performance of CT colonography in polyp detection [51]. MRE with colonic preparation offers the advantage of obtaining comprehensive information on both small- and large-bowel involvement in patients with IBD during a single session, without the technical challenges associated with MRC. These technical challenges include the need for retrograde filling of the entire colon with fluid using such methods as a Foley catheter or rectal tub, which can be uncomfortable for patients. Furthermore, MRC disallows the visualisation of the small bowel, leading MRE to serve as an alternative diagnostic method for patients experiencing flare-ups of UC when colonoscopy is contraindicated. This may occur in situations where there is an elevated risk of certain toxic or perforation conditions. In specific clinical scenarios, technical limitations, such as luminal strictures or a severe dolichocolon, can pose challenges to achieving a complete or successful colonoscopy. In such cases, MRE emerges as a valuable alternative modality for diagnostic evaluation. Moreover, MRE can be utilised as a valuable tool in patients with refractory UC or suspected terminal ileum involvement, aiding clinicians in excluding CD [52]. In addition, MRI offers the potential to delineate the specific pattern of colonic involvement and facilitate the identification of small intestine or extraintestinal pathological conditions in patients with UC, tasks that extend beyond the reach of colonoscopy visualisation. 

MRI exhibits many inherent advantages over other imaging modalities, including CT and X-ray, rendering it highly desirable for the evaluation of IBD. One of the primary advantages of MRI in evaluating IBD is the absence of ionising radiation exposure to patients. Due to the necessity for the frequent reassessment of disease activity in patients with IBD and concerns regarding cumulative exposure to ionising radiation from imaging modalities, there is a growing preference for MRI over computed tomography enterography (CTE) [39,53]. Furthermore, compared to CT, MRE offers superior soft tissue contrast, even in the absence of intravenous contrast administration. This characteristic is particularly valuable for identifying mesenteric inflammatory changes and bowel wall oedema in the evaluation of IBD. Several studies have consistently shown a high level of accuracy of MRE in comparison to colonoscopy and CTE in the detection of active disease in patients with IBD [54,55]. However, MRE has several limitations, including a prolonged scan duration, often requiring sedation in young or neurologically impaired patients, and the inability to perform the procedure in individuals with MRI-incompatible devices or metallic foreign bodies [56]. Table 1 summarises the pros and cons of medical imaging modalities for IBD. 

**Table 1:** Pros and cons of medical imaging modalities for IBD.

Imaging Modality	Pros	Cons
MRI	Safe and non-invasive No exposure to harmful ionizing radiation Ability to image the bowel repeatedly over time Exceptional soft tissue contrast enables superior evaluation of disease activity and the identification of penetrating disease complications Able to identify both luminal and extraluminal abnormalities [56]	Long image acquisition, which often needs sedation for neurologically impaired and young patients Need bowel preparation Use contrast agent (if requested) Patients with metallic foreign bodies or MRI-incompatible devices cannot perform MRI [56]
US	Safe Non-invasive Lacks radiation exposure Portable [56]	Operator-dependent Assessment of deep bowel loops is hindered by acoustic absorbance in tissues, limiting the effectiveness of ultrasound [57] The sensitivity remains uncertain or inadequately established in pregnancy [56]
CT	Short image acquisition The capacity to image paediatric patients without requiring sedation CTE exhibits high sensitivity and specificity in detecting active inflammation within the small intestine, accompanied by improved interobserver agreement and more consistent image quality, including enhanced spatial and contrast resolution, when compared to MRE [56]	The potential exposure of patients to ionizing radiation
DWI	Short scan time Safe and non-invasive sequence Quantitative and qualitative analysis No need for bowel preparation or contrast agents [56]	Sensitive to motion artifact T2 shine artifact Poor anatomical visualization

## 4. Assessing Disease Activity in UC Using DWI

MRI platforms have shown potential as a valuable tool for evaluating disease activity and treatment response in patients with UC. The Nancy Score and the Clermont Score are two commonly used disease grading systems that make use of DWI. These scoring methods have gained popularity and are widely utilised in clinical practice for evaluating the severity or activity of various diseases [24,58]. The Nancy score, a clinical assessment tool utilized in the evaluation of Crohn’s disease, ranges from 0 to 36. According to the Simplified Endoscopic Score for Crohn’s Disease (SES-CD), a Nancy score greater than two is closely associated with the presence of endoscopic inflammation, as indicated by a statistically significant positive correlation (r = 0.539, *p* = 0.001) [24]. Nancy score, for example, was designed to eliminate oral bowel preparation and has proven to be a reliable technique to assess colonic inflammation, making it a valuable tool in UC [59]. In UC, data on DWI are scarce. Table 2 summarises available studies on DWI assessing disease activity and treatment response in UC. 

**Figure 1:**
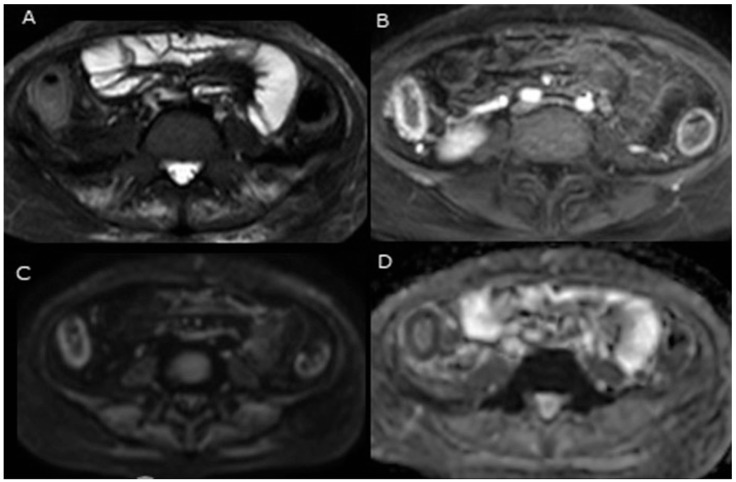
A Thirteen-year-old girl diagnosed with moderate activity of UC. On an axial T2-weighted image with fat saturation (**A**), noticeable findings included wall thickening measuring 7 mm and moderate hyperintensity (indicative of mural oedema) in the ascending and descending colon. An axial T1 post-contrast weighted image with fat saturation (**B**) exhibited pronounced mural enhancement. Axial DWI (**C**) and the corresponding ADC map (**D**) demonstrated conspicuous diffusion restriction within the colonic wall (figure taken from Figure 3 of Saleh et al. [60]).

Over the past decade, DWI has been proposed as a potential method for assessing disease activity in patients with IBD. The study by Buisson et al. investigated the relationship between quantitative DWI parameters and the endoscopic features of inflammatory ulcerations in patients with IBD. The findings of their study found a significant inverse correlation between the ADC values and the depth and size of the inflammatory ulcerations. Areas with deeper and more extensive ulcerative lesions exhibited lower ADC values, suggesting a restricted diffusion of water molecules within the inflamed and disrupted intestinal wall. In another study, findings by the same group demonstrated that the quantitative analysis of DWI-derived parameters, specifically the ADC, exhibits a high degree of accuracy in the detection and assessment of inflammatory activity in patients with colonic CD [58]. DWI can be also used to determine the inflamed portion in UC. For instance, in one study, Aoyagi et al. [22] assessed the feasibility of utilising DWI as a diagnostic tool for detecting bowel inflammation and investigated the alterations in ADC values specifically within the inflamed bowel segments of seven patients diagnosed with UC. The resected specimens were examined histopathologically to determine the degree of inflammation and the presence of malignancy. They found that the mean ADC value of the normal colorectal tissue was significantly higher than that of active UC lesions. Moreover, the average ADC values of the active lesions were found to be significantly lower when compared to those of the healing lesions [22]. The study concluded that the ADC values could potentially serve as valuable data in the staging of UC. Another prospective study was conducted to evaluate the efficacy of MRE and DWI in detecting the activity of 24 paediatric IBD patients (9 CD and 15 UC) and their correlation with endoscopic and clinical activity scores [60]. These patients underwent DWI and MRE, as well as a clinical examination and ileocolonoscopy within a period of under 2 weeks. Prior to the MRI examination, all participants underwent a fasting period of 4–6 h, followed by the ingestion of a hyperosmolar oral contrast agent to achieve adequate distension of the small bowel. The MES and MRE scores were calculated for UC patients, demonstrating a significant positive correlation among UC cases between MRE severity and MES [60]. Haustral loss was observed in eight cases of UC, while it was not detected in patients with CD. The descriptive case of UC is depicted in Figure 1. This figure was taken from Figure 3 in Saleh et al. [60].

In another observational study involving 35 patients with UC, the accuracy of DWI-MRI in assessing disease activity was investigated. The DWI used a fixed diffusion factor bb of 600 s/mm², resulting in two sets of images: one corresponding to the expected b value and another at b = 0. The study was conducted without the use of oral or rectal preparations and fasting, and the results showed that endoscopic activity, as determined by a segmental Nancy score greater than 1, was accurately detected by DWI-MRI with a sensitivity of 89.4% and specificity of 86.7%. The area under the receiver operating characteristic curve (AUROC) was 0.92, indicating a high diagnostic accuracy [24]. The segmental Nancy scores and total Nancy scores demonstrated significant agreement with the segmental modified endoscopic Baron scores and the total modified endoscopic Baron scores, respectively. In the study, DWI hyperintensity was found to be an independent predictor of endoscopic activity, with an odds ratio (OR) of 13.26 and AUROC of 0.854. Furthermore, the study revealed that DWI hyperintensity exhibited comparable accuracy to gadolinium-based contrast agent enhancement in detecting endoscopic inflammation [24]. Yu et al. [61] conducted a subsequent confirmation of these findings was conducted in a later study, and their results demonstrated that performing DWI at a b value of 800 s/mm^2^ improved the accuracy of DWI hyperintensity in detecting endoscopic inflammation. The sensitivity and specificity of DWI hyperintensity were reported as 93% and 79.3%, respectively, with an area under the curve (AUC) of 0.867 [61]. The study additionally identified a specific threshold ADC value of 2.18 × 10^−3^ mm^2^/s that consistently detected endoscopic inflammation, with a sensitivity of 89.7% and specificity of 80.3%. Few studies reported differences between the ADC values of inflamed and normal bowel segments of patients with UC [61,62]. One study conducted by Kılıçkesmez et al. aimed to assess the efficacy of DW-MRI in the assessment of disease activity in 28 UC patients [23]. They found that the ADC values of the rectum exhibited statistically significant differences (*p* = 0.009) among patients in the active phase (1.08 ± 0.14 × 10^−3^ mm^2^/s), sub-acute phase (1.13 ± 0.23 × 10^−3^ mm^2^/s) and remission phase (1.29 ± 0.17 × 10^−3^ mm^2^/s) of the disease [23]. In another study, the average ADC value of endoscopically confirmed inflamed bowel segments was determined to be 1.56 ± 0.58 × 10^−3^ mm^2^/s, whereas it was 2.63 ± 0.46 × 10^−3^ mm^2^/s in normal bowel segments, indicating a statistically significant difference [61].

Another recent study was conducted to determine the efficacy of IVIM-DWI with 10 b-values (0–900 s/mm^2^) in assessing inflammatory activity in 20 patients with UC [25], where the gold standard was histopathological and endoscopic scoring. They also compared the endoscopic score with the IVIM-MRI-derived parameters in 56 bowel segments. They found no significant correlation between f, D or D* and the MES, as active and non-active diseases were compared endoscopically. The researchers also compared histopathological data and the IVIM-derived parameters in 34 bowel segments, and they found a significant difference between moderate-to-severe inflammation (grades 3–5) and non-active or less-active diseases (grades 0–2). These findings underscore the sensitivity, reproducibility and simplicity of utilising the Nancy score for assessing therapeutic efficacy. The researchers concluded that the IVIM perfusion fraction is correlated with UC activity and may serve as an emerging tool for evaluating inflammatory activity.

## 5. Monitoring UC Using DWI

Resistance to anti-tumour necrosis factor (anti-TNF) therapy presents a significant barrier to the management of UC, leading to considerable treatment expenses. Imaging modalities present a promising alternative for monitoring ulcerative colitis compared to invasive current procedures. However, they are not yet deemed adequate for evaluating mucosal healing, despite the advancement of novel techniques [63]. A study involving a small cohort of CD patients (n = 48) found that the MRE disease index is a viable tool for assessing mucosal healing [64]. Yet, MRI has shown potential as a valuable tool for evaluating mucosal healing and treatment response in patients with IBD. In a recent study conducted on a cohort of patients with UC, the accuracy of the DWI Nancy score was evaluated to assess mucosal healing, defined by an MES ranging from 0 to 1 [59]. In addition, the study examined the treatment response in a subgroup of subjects with active UC. Laurent et al. [59] prospectively analysed 29 UC patients treated with various medications who had undergone MRE before treatment induction. Both the MES and the Nancy score were used to evaluate disease activity. Mucosal healing was consistently identified by a Nancy score below seven, demonstrating a sensitivity of 75% and specificity of 67%. The AUROC was calculated as 0.72, indicating moderate accuracy, and the study found that the Nancy score exhibits good reliability, as indicated by an intraclass correlation coefficient (ICC) of 0.63 [59]. Among patients who achieved mucosal healing, both the MES and the Nancy score exhibited significant reductions, where the latter decreased from 18.2 at baseline to 3 at revaluation and the former decreased from 2.4 at baseline to 0.6 at revaluation. Similarly, MRC demonstrated high accuracy in diagnosing disease severity and activity in UC [34]. Further research is necessary to validate imaging modalities and indexes, as well as to establish their correlations with long-term disease outcomes. 

Overall, these findings illustrate that DWI holds promise for accurately discerning therapeutic efficacy in patients with UC and may serve a predictive role in determining disease courses. Compared to conventional MRE sequences, DWI offers advantages in terms of expedited imaging, a simplified procedure, reduced invasiveness and enhanced patient tolerance, owing to its independence from intravenous contrast agents and lack of need for bowel preparation or fasting for colonic assessment.

**Table 2:** Some available studies using DWI in UC.

Reference	Subject (Bowel Segments) Type of Study	Standard Ref. b-Values s/mm^2^	Bowel Preparation	Findings
Laurent et al. [59]	29 ulcerative colitis (UC) patients Prospective, observational study	Colonoscopy 0 and 600	No	Nancy score < 7 indicated a sensitivity, specificity and accuracy (75%, 67% and 73%, respectively, in the diagnosis of mucosal healing (area under the receiver operating characteristic curve. AUROC]): 0.72; 95% confidence interval [CI], 0.56–0.88; *p* = 0.0063). In patients who achieved MH, both the Mayo endoscopic subscore and Nancy score showed significant reductions. Specifically, the Mayo endoscopic subscore decreased from 2.4 at baseline to 0.6 at re-evaluation (*p* = 0.02) and the Nancy score decreased from 18.2 at baseline to 3 at re-evaluation (*p* = 0.006). Conversely, in active patients at reassessment, the Nancy score did not exhibit significant changes. The association between the total Nancy score and the Mayo Endoscopic Subscore was not good at the first assessment [r = 0.10; *p* = 0.61] but was good at the second assessment [r = 0.62; *p* < 0.005]. For the total Nancy score, the intra-class correlation coefficient for inter–intra-observer agreements at baseline and reassessment were 0.89 [0.76–0.95]; 0.79 [0.60–0.90] and 0.99 [0.97–0.99]; 0.99 [0.98–0.99], respectively. The study concluded that the Nancy score is a highly responsive, reliable tool for assessing treatment response in UC patients.
Oussalah et al. [24]	28 UC patients (105 segments) Prospective study	Colonoscopy 0 and 600	No	The presence of hyperintensity on diffusion-weighted imaging (DWI) demonstrates a significant predictive value for endoscopic inflammation, as indicated by an odds ratio (OR) of 13.26 (95% CI: 3.6–48.93) and an area under the curve (AUC) of 0.854 (*p* = 0.0001). A total segmental magnetic resonance (MR) score of greater than 1 meant a sensitivity, specificity and AUROC of 89.47%, 86.67% and 0.920, respectively. The proposed total MR score correlated with the Walmsley index (r = 0.678, *p* < 0.0001) and the total modified Baron score (r = 0.813, *p* = 0.0001) in those patients. The presence of a DWI hyperintensity (DWI-HI) showed a sensitivity of 90.79% and a specificity of 80% for detecting endoscopic inflammation, with an AUROC of 0.854 (*p* = 0.0001) In detecting endoscopic inflammation, the DWI hyperintensity had the same accuracy as gadolinium-based contrast agent enhancement. The study concluded that the MR-DWI colonography rectal preparation or without oral could represent a non-invasive tool in evaluating colonic inflammation in UC.
Jesuratnam-Nielsen et al. [65].	25 UC patients (No available) Prospective study	MRE 0, 100, 200, 500, 700, 800 and 1000	No	The findings of the study reported that the DWI’s sensitivity, specificity and accuracy ranged from 0 to 52%, 83 to 94% and 76 to 92%, respectively. DWI-MRI in the colon exhibited significant false-positive findings attributed to the T2 shine-through phenomenon.
Yu et al. [61]	20 UC patients (100 segments) Prospective observational study	Colonoscopy 0, 400, 600, 800, and 1000	No	DWI hyperintensity at a b-value of 800 s/mm^2^ reliably indicated the presence of endoscopic colonic inflammation, with a sensitivity of 93.0%, specificity of 79.3% and statistically significant AUC of 0.867 (*p* < 0.0001). The segmental MR score (MR-score-S) exhibited a significant positive correlation with the segmental modified Baron score (Baron-S) (r = 0.761, *p* < 0.0001). Similarly, the total magnetic resonance score (MR-score-T) demonstrated a strong positive correlation with the total modified Baron score (Baron-T) (r = 0.875, *p* < 0.0001). An MR-score-S greater than 1 was found to be indicative of endoscopic colonic inflammation, with a sensitivity and specificity of 85.9% and 82.8%, respectively, and an AUC of 0.929 (*p* < 0.0001). DWI hyperintensity at a b-value of 800 s/mm^2^ demonstrated significantly higher diagnostic accuracy compared to b-values of 400, 600 and 1000 s/mm^2^. The study determined that an ADC value of 2.18 × 10⁻³ mm^2^/s effectively identifies endoscopic inflammation, demonstrating sensitivity and specificity of 89.7% and 80.3%, respectively. For DWI hyperintensity, the inter-observer agreements across different b values were consistent, with kappa values ranging from 0.719 to 0.825. The ADC measurements obtained by the two radiologists were compared, revealing a Pearson’s correlation coefficient of 0.886 (*p* < 0.001), which indicates a high level of inter-observer agreement. They concluded that the integration of DWI with conventional MRI, without the need for bowel preparation, offers a quantitative approach to distinguishing actively inflamed intestinal segments from normal mucosa, enabling the detection of UC.
Podgórska et al. [25]	20 UC patients Prospective study	Endoscopic and histopathological scoring 0, 10, 30, 50, 75, 100, 150, 200, 500 and 900	Yes	The analysis did not reveal any statistically significant correlation between the Mayo endoscopic subscore and the measured parameters of D*, D or f as active and non-active diseases were compared endoscopically. Statistical analyses found that there were statistically significant differences in the parameters of D, between cases characterized by histopathologically classified inactive or mild disease activity and those with moderate to severe disease activity (respectively, mean = 1.34 10^−3^ mm^2^ /s and mean = 1.07 × 10^−3^ mm^2^ /s, *p* = 0.0083; AUC = 0.735, sensitivity 0.91, specificity 0.54, accuracy 0.66 for cut-off value 1.24 × 10^−3^ mm^2^ /s and mean = 0.19 and mean = 0.28, *p* = 0.024; AUC = 0.723, sensitivity 0.82, specificity 0.59, accuracy 0.67 for a 0.185 cut-off value). However, for D* no significant difference was found. This study did not analyse ADC.
Kılıçkesmez et al. [23].	28 UC patients Prospective study	Colonoscopy 0, 500 and 1000	No	The ADC values of the sigmoid colon did not significantly differ among patients in the active, subacute, and remission phases of UC (*p* = 0.472). The ADC values of the rectum were different between patients in the remission, subacute and active phases. The rectum ADC values of the patients in remission were higher than the rectum ADC values of patients in subacute (*p* = 0.007) and the active (*p* = 0.009) phases and were similar in patients in the subacute and active phases of the disease (*p* > 0.05). The study found that higher disease activity is associated with lower ADC values.

## 6. Discussion and Conclusions 

In this review, few studies were identified to have adopted DWI-MRI in patients with UC. In the context of the ‘treat-to-target’ approach, there is an increasing need for accessible, feasible and non-invasive tools to monitor patients with UC. This urgency stems from the desire to alleviate the burdens associated with invasive procedures, such as colonoscopies. MRE has emerged as a potential substitute for colonoscopy in UC. However, the MRE involves certain requirements, such as expanding the intestinal lumen, fasting, preparing the bowel and administering a contrast agent intravenously. DWI-MRI overcomes some of these confines, as neither fasting nor preparation is required for the study of the colon, and the administration of a contrast agent intravenously, specifically gadolinium, is not mandatory for the technique [62]. 

In most published studies focusing on the use of DWI in CD, oral contrast agents have been commonly used to expand the bowel lumen for optimal imaging [38,66], which is inconsistent with our findings in this review. In this review, most studies conducted DWI examinations in patients with UC without administering oral fluid contrast agents [24,61,65]. Bowel preparation was linked to the occurrence of acute exacerbation in individuals with UC, so utilising such diagnostic methods as DWI, which does not necessitate bowel preparation, has the potential to circumvent the complications of acute exacerbation in patients with UC. Therefore, the proposed technique can be seamlessly integrated into conventional MR examination protocols due to its short duration. The Nancy score exhibits an intuitive nature, as it does not rely on a complex mathematical formula for calculation. Moreover, it circumvents the use of ADC values, which can be subject to poor reproducibility and reliability concerns. Moreover, the qualitative evaluation of signal intensity within the bowel wall on DWI is incorporated into the Nancy scoring system, which has been reported to exhibit a good correlation with endoscopic findings and treatment-response assessment in individuals with UC as discussed in Table 2 [24,59,61]. 

The integration of conventional MRI sequences with DWI holds the potential for evaluating colonic inflammation in individuals diagnosed with UC, even in the absence of rectal preparation or oral contrast administration. The DWI sequence showed comparable [61] or higher [24] accuracy to post-contrast sequences in detecting endoscopic inflammation in UC. These results suggest that the DWI sequence has the potential to serve as a substitute for gadolinium injection in identifying inflammatory colonic segments in UC. Moreover, the findings from these studies provide additional evidence supporting the feasibility of conducting contrast-free MRE, thereby eliminating the risks associated with gadolinium-based contrast agents, including allergic reactions, renal impairment and potential long-term deposition in the central nervous system.

Several studies in this review employed the b factor at 600 s/mm² using 1.5 T as this value represented the optimal balance between signal-to-noise ratio and lesion detection sensitivity [24,59]. However, in another study [61], DWI hyperintensity at a b-value of 800 s/mm^2^ on a 3T scanner exhibited the highest diagnostic efficiency for detecting colonic inflammation in patients with UC. The variation in b values between these studies’ findings may be attributed to the uniformity of the primary magnetic field and differences in field strength.

In terms of monitoring treatment response, more longitudinal follow-up studies that directly compare imaging data before and after biological therapy in the same patient are required which can provide a more accurate assessment of the significance of DWI in monitoring inflammatory activity in UC. Most existing evidence is from cross-sectional studies, and there is only one published study on longitudinal monitoring [59]. In CD, few studies have been conducted to investigate the role of ADC values in identifying bowel inflammation and assessing therapeutic response undergoing biologic therapy [67,68]. None of the studies in this review explore the role of ADC in evaluating the biological response. 

Some limitations should be acknowledged. Variations in DW-MRI protocols, such as acquisition techniques and b-values, across different studies may impact the comparability of results. Standardisation of protocols is thus necessary to ensure reliable and consistent assessments. In addition, DW-MRI may be limited in accuracy in differentiating inflammation from fibrosis, which is an important consideration for treatment decisions in IBD in general.

In conclusion, DWI-MRI is a fast, less invasive procedure than conventional MRE. Using DWI, especially in combination with restricted diffusion with ADC, provides additional diagnostic value to conventional MRE for differentiating accurately between active and inactive IBD without the need for contrast administration. This technique may play a significant role in diagnosing UC. Further studies are required to validate DWI techniques, indices and relationships with long-term disease outcomes.
